# *Exon1* and *-116 C/G* Promoter Polymorphism on the X-Box DNA Binding *Protein- 1* Gene is not Associated with Breast Cancer among Jordanian Women

**DOI:** 10.31557/APJCP.2019.20.9.2739

**Published:** 2019

**Authors:** Lulu H Alsheikh Hussein, Ahmad M Khalil, Ahmad Y Alghadi, Abed Alkarem Abu Alhaija

**Affiliations:** *Department of Biological Sciences, Yarmouk University, Irbid, Jordan.*

**Keywords:** −116 C/G polymorphism, breast cancer, SNPs, XBP1gene

## Abstract

**Background:**

Human *X -box binding protein 1* (*XBP1*), a critical gene in the endoplasmic reticulum stress response, is located on chromosome 22q12, which has been linked with the pathogenesis of many diseases, particularly cancers such as breast cancer (BC). Single nucleotide polymorphisms (SNPs) in the* XBP1* gene can alter structure and function of the gene. In this study, polymorphism in the promoter region and exon1 of the gene *XBP1 *and its association with BC in Jordanian women was investigated.

**Methods:**

Polymorphism in the promoter and exon1 of *XBP1* was analyzed in 100 subjects (controls: n=40; BC patients=60). *−116 C/G* SNP was genotyped by Polymerase Chain Reaction (PCR)-sequence specific primer technique. The odd ratios (ORs) at 95% confidence intervals (CIs) were computed to assess the strength of this association.

**Results:**

The three genotypes of the SNP (GG, GC, CC) and their allelic frequencies have nonsignificant differences between patients and control group. It was noticed that the frequencies of the mutant allele (G) were (75.8% versus 24.2%)) in the patients and control groups, respectively, while those of the normal allele (C) were (67.5% versus 32.5%). *XBP1* (-116 G→C) G allele did not show significant association with BC risk (confidence interval = 0.3534- 1.2395, odds ratio = 0.6619, P= 0.197). Moreover, there were no significant mutations in the* XBP1* exon1 neither in BC subjects nor control subjects.

**Conclusions:**

This is the first study to evaluate the effect of polymorphism in the promoter and exon1 of *XBP1* gene in the pathogenesis of BC in Jordanian women. The results do not support a role for polymorphism in development of BC and further studies with a larger sample size and detailed data should be performed in other populations.

## Introduction

Breast cancer (BC) is considered to be the most common carcinoma and the second leading cause of cancer-related death in women throughout the world (Siegel et al., 2018). BC is a public health problem in most countries as stated by the World Health Organization (WHO, 2008). Official Jordanian statistics indicated that BC was the most common of all cancers in women, with 994 diagnoses in 2012 (37.3 percent) (Directorate of Information Studies and Research, 2005; Jordan Cancer Registry (JCR, 2012). 

The growing prevalence of BC incriminates many factors in development of BC such as heredity, age, reproductive history, oral contraceptives, breast density, parity, breastfeeding, alcohol use, body mass index (BMI), exercise, hormone replacement therapy, and menstrual history (Khan et al., 2010; Abbad et al., 2018; Lilyquist et al., 2018). The interaction between inherited mutated genes and environmental factors is believed to play a crucial role in cancer development (Khan et al., 2010). Cancer-related deaths are due to modifiable factors such as smoking, alcohol consumption, low fruit and vegetable intake and a high fat-diet (Danaei et al., 2005; Khan et al., 2010; Kamińska et al., 2015; Al Qadire et al., 2018). In this context, it is estimated that 5-10% of all breast carcinomas are inherited.* BRCA1* and *BRCA2* genes are responsible for 3-8% of all BCs and for 15-20% of the inherited BC (Pilato et al., 2011; Easton et al., 2015). 

There are also other gene mutations besides BRCA that could increase the risk of BC. Researchers have discovered, and are continuing to discover, other abnormal genes e.g.* BRIP1*, *CDH1*, *PTEN*, *TP53*, and the *STK11* genes that are less common than *BRCA1*, *BRCA2*, and *PALB2* but also can raise BC risk in each population (Easton et al., 2015; Lincoln et al., 2015; Shiovitz and Korde, 2015; Daly et al., 2017; Abbad et al., 2018; Lilyquist et al., 2018; Momozawa et al., 2018). *BARD1*, *BRCA1*, *BRCA2*, *PALB2*, and *RAD51D* genes were associated with high risk for triple-negative BC and greater than 20 percent lifetime risk for overall BC among Caucasians (Shimelis et al., 2018). They observed a similar trend among African Americans population. Prediction models suggest that there are unlikely to be additional yet to be identified as high-penetrance genes. 

The human* X-box binding protein-1* (*XBP1*) gene is located on chromosome 22q12.1 (Liou et al., 1990). *XBP1 *is a major component of the unfolded protein response (UPR) and is essential for maintaining protein homeostasis and reducing cellular stresses (Hetz et al., 2013). Evidence has emerged that high expression of the nuclear *XBP1 *gene plays a role in BC development (Bertucci et al., 2000; Ding et al., 2004; Scriven et al., 2009; Chen et al., 2014; Ming et al., 2015; Wang et al., 2017; McGrath et al., 2018). 

**Figure 1:**
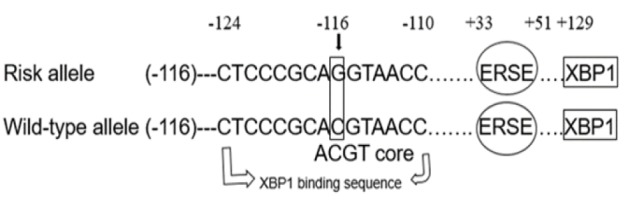
-116C→G Polymorphism in *XBP1* Gene Promoter. -116C→G Abolishes the ACGT Core Sequence. Numbers indicate the nucleotide position from the transcription site. ERSE, Endoplasmic reticulum stress response element

Since almost all the available data are from the population of the European descendent (Lilyquist et al., 2018; Mavaddat et al, 2019), it is unclear whether clinical interpretations are generally applicable to other populations. The finding of Hoffman et al., (2019) highlighted the utility of performing additional searches for genetic variants for BC in non-European populations. In Jordan, 30.5% of the BC cases were diagnosed at stage III and IV, thus great efforts should be made to improve this percentage through a good surveillance system and screening programmes for high-risk groups. To date, 18% of the familial risk of BC can be explained by common single nucleotide polymorphisms (SNPs) (Lilyquist et al., 2018). Single nucleotide polymorphisms (SNPs) risk information can potentially improve the accuracy of BC risk prediction (Fung et al., 2019). The aim of this study was to evaluate the relationship between exon 1 and promoter (*-116C/G*) polymorphism (Figure 1) in the *XBP1 *gene and BC risk in Jordanian women. 

## Materials and Methods

*Study population*

This study was conducted in a large hospital; King Abdullah University Hospital (North of Jordan). The sample consisted of two genetically unrelated groups: 60 BC patients and 40 healthy; cancer free - control. The age of the two groups was matched (55±5 years). None of the patients or healthy controls was cigarette smoker, alcohol or drug user. BC diagnosis was confirmed by standard laboratory and histopathology reports. Detailed information on histopathological variables and clinical data were available in a database. Approval to undertake this study was confirmed by the Ethics Committee of the King Abdullah University Hospital. All procedures were conducted according to the Declaration of Helsinki Principles. Written informed consent was obtained from each recruited subject. 

*Sequence Analysis and SNP Genotyping*

Under complete aseptic conditions, 5 ml of venous blood were collected from each participant in a sterile EDTA treated tube. DNA was extracted from peripheral blood samples using a Wizard Genomic DNA Purification Kit (Promega, USA) according to the manufacturer instructions. All the primers and restriction enzyme used in this study were designed manually. The location and fidelity of restriction enzyme and primers sequence were checked using the following software: 

- http://primer3.ut.ee/,https://genome.ucsc.edu/, 

- http://ensembl.org/Homo_sapiens/Gene, and

- http://www.labtools.us/nebcutter-v2-0/ 

The primers for *rs2269577 SNP* were 5′-GTTTCAGGACCGTGGCTATG-3′ (Forward primer) and 5′-TCAGTCTGGAAAGCTCTCGG-3′ (Reversed primer). We designed primers flanking the exon 1, as well as 186 bp upstream (containing putative regulatory elements) and 48 bp from intron 1 sequences of XBP1 gene. Exon 1: 5´- GCGGAAAATGACCCCAAGTA-3´ (Forward primer) and 5´- CCTAGTCCCGGCTTCAGATC-3´ (Reversed primer). A total of 50 ng genomic DNA was amplified in a 25 µL final volume PCR reaction containing 0.4 µM of each primer and 12.5µL of the SYBR Green PCR master mix (GoTaq^®^Green Master Mix, Promega, USA). Amplification was performed at 95°C for 5 min with an initial denaturation, followed by 35 cycles of 95°C for 30 Sec, 52°C for 30 Sec, and 72°C for 30 Sec and a final extension of 5 min at 72°C. The amplified fragment of 190 bp of PCR products were digested with the BstEII restriction enzyme.

**Table 1:** Chi-squared Comparisons between Genotype Distributions of XBP1 -116C/G in the Breast Cancer Patients and Control Group

	Genotype	Observed	Expected	Genotype	Observed	Expected
(-116 C→G)	GG	37	33	GG	18	22
	GC	17	21	GC	18	14
	CC	6	6	CC	4	6
*p-value*	0.210					

**Table 2:** Distribution of XBP1 SNP* (-116 G→C) Genotype and Allele Frequencies among Breast Cancer Patients and Control Subjects

XBP1* (-116 G→C) Genotype	Breast Cancer Number (%)	Control Number (%)	OR*	95% CI*	p-Value <0.05
GG	37 (61.7%)	18 (45%)	0.73	0.18-2.91	0.655
GC	17 (28.3%)	18 (45%)	0.63	0.15-2.63	0.525
CC	6 (10%)	4 (10%)	1.05	0.91-5.20	0.079
Allele Frequencies					
G	91 (75.8%)	54 (67.5%)			
C	29 (24.2%)	26 (32.5%)	0.6619	0.3534-1.2395	0.197

* SNP, Single nucleotide polymorphism; XBP1, X-box binding protein-1; OR, Odds ratio; CI, confidence interval

The products were amplified by polymerase chain reactions (PCR) with touchdown program (95°C for 5 min; 30 cycles of 95°C for 30 s, 60°C for 30s, 72°C for 30 s, 72°C for 5 min). 

*Statistical analysis*

The presence of Hardy–Weinberg equilibrium was examined by using the *X*^2^ test for goodness of fit. Genotype and allele distributions between both groups were analyzed by the *X*^2^ test for independence. Chi square test, odds ratio (OR) with 95% confidence level (CI) estimation were performed in order to measure the association between *XBP1* polymorphisms and BC risk. The level of statistical significance was set at P<0.05.

## Results

As shown in Table 1, genotype distribution for the SNP -116 C→G was within the Hardy–Weinberg equilibrium in both groups (P=0.210). Table 2 presents the distribution of *XBP1* SNP (-116 G→C) genotype and allele frequencies among BC patients and control subjects. There were no statistically significant associations between observed and expected numbers of 116 C→G genotypes among BC patients and control. Results indicate that the highest genotypic frequency in patients and controls groups was (61.7% versus 45%), respectively, while the lowest frequency (10%) was recorded for the genotype (CC) in both groups. The OR and CI records for GG, GC, CC genotypes were (0.73, 0.18-2.91), (0.63, 0.150-2.63) and (1.05, 0.91-5.20), respectively. The results show that the risk-associated G allele has nonsignificant difference than non-risk C allele; 75.8% versus 67.5% and 24.2% versus 32.5%, in the patient and control groups, respectively with OR=0.662, CI=.354-1.240 and P˃0.05. Furthermore, sequencing analysis revealed no remarkable mutations in exon1 of the* XBP1 *gene.

## Discussion

The present study is the first report to examine the risk factors associated with *XBP1 *SNP in BC progression in Jordan. Our statistical analysis demonstrated that the G/G genotype of the* SNP rs2269578* was not significantly associated BC compared with the homozygous C/C genotype (P = 0.073, odds ratio = 0.662). This SNP is located in the promoter of the gene* XBP1*, affecting the binding site of the gene. We were unable of detecting any remarkable mutation in exon1 of *XBP1* gene. The power of this study was limited by small sample size. Therefore, the lack of association could be due to the small number of patients included in the analysis. This reflects the difficulty in enrolling untreated non-smoking patients which is necessary to avoid confounding effects that could influence the sensitive parameters being investigated in the study. SNP studies require thousands of cases and controls to have sufficient power to appreciate a change in risk, as individual alleles may be relatively common and even found in a majority of the population (Stratton and Rahman, 2008). There are no studies to suggest that that the use of single SNPs is informative for evaluating the risk of diseases, thus has quite small impact on clinical improvements in human health care outcomes (Medical Policy Manual, 2019). Most of the BC susceptibility SNPs reside in intergenic or intronic regions of the genome and counter to early expectations, do not generally impact protein-coding regions (Edwards et al., 2013). Due to small number of investigated population we were unable of carrying out a subanalysis stratifying BC patients such as SNP association with stage of cancer, family history, overweight, hormone replacement therapy or radiotherapy. 

Studies combining common variants together may provide some insight into individual risks of BC. A number of SNPs associated with BC have been found at a high level of statistical significance and validated in two or more large, independent studies (Thomas et al., 2009; Ahmad et al., 2009, Kim et al., 2012). Stratification of women according to their risk of breast cancer based on polygenic risk scores could improve screening and prevention strategies (Mavaddat et al., 2019). Earlier studies using polygenic risk scores based on larger numbers of SNPs have successfully demonstrated an ability to stratify or individualize BC risk in a number of populations (Gail, 2008). A more recent OncoArray study suggested that all of the BC susceptibility markers to date explain up to 18% of familial relative risk (Michailidou et al., 2017). One way to identify SNP function and a potential target gene is to evaluate expression quantitative trait loci (eQTLs) to determine whether a SNP is impacting gene expression. It should be recalled that our study was not designed to evaluate the potential gene expression changes in relation to SNP. However, eQTL studies can be limited by the normal tissue that is available (Lonsdale et al., 2013). Transcriptome-wide association studies (TWAS) have been used to analyze both SNPs and gene expression to identify novel BC susceptibility genes and loci. By pairing genotyping data with gene expression data, SNPs that can predict gene expression can be selected (Lilyquist et al., 2018).

In conclusion, the clinical implication of our finding is that the* -116C/G *polymorphism of *XBP1* promoter may not be relevant to susceptibility to BC in Jordanian women. Moreover, analysis of exon1 of the* XBP1* only does not negate the association with BC, so this outcome cannot be generalized to other populations. The study provides important data to guide prospective genetic testing for BC susceptibility genes including the* 22q12.1* locus in greater depth in an expanded sample size of BC cases and controls in other populations using improved methods. 
